# Supplementary figures and images for: A developmental gradient of COUP-TFI expression regulates the relative size of hippocampus dorsal and ventral subregions
Source: PLoS Biol. 2025 Aug 25;23(8):e3003355. doi: 10.1371/journal.pbio.3003355 (PMC12396750; doi:10.1371/journal.pbio.3003355)

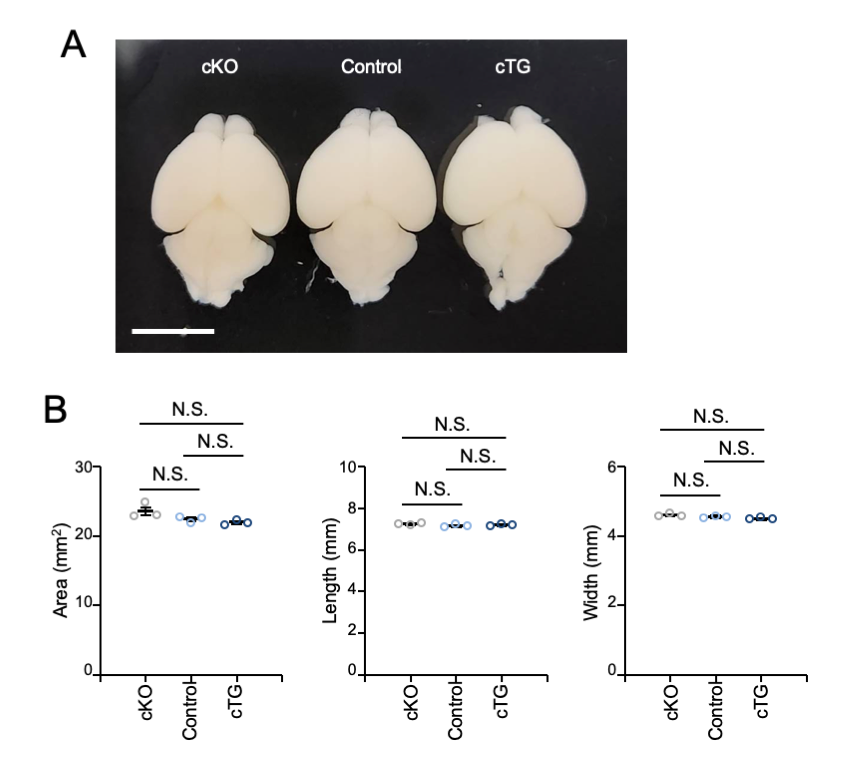

Supplement: S1 Fig — (A) Dorsal views of the brain from control and COUP-TFI mutant mice at P7. (B) Comparisons of brain surface area, length, and width in control and COUP-TFI mutant mice revealed no significant differences (N.S.). Scale bar, 5 mm. Statistical analyses were performed using Student t test. The data underlying this figure can be found in S1 Data. (S1_Fig.TIFF) [file pbio.3003355.s001.tiff]

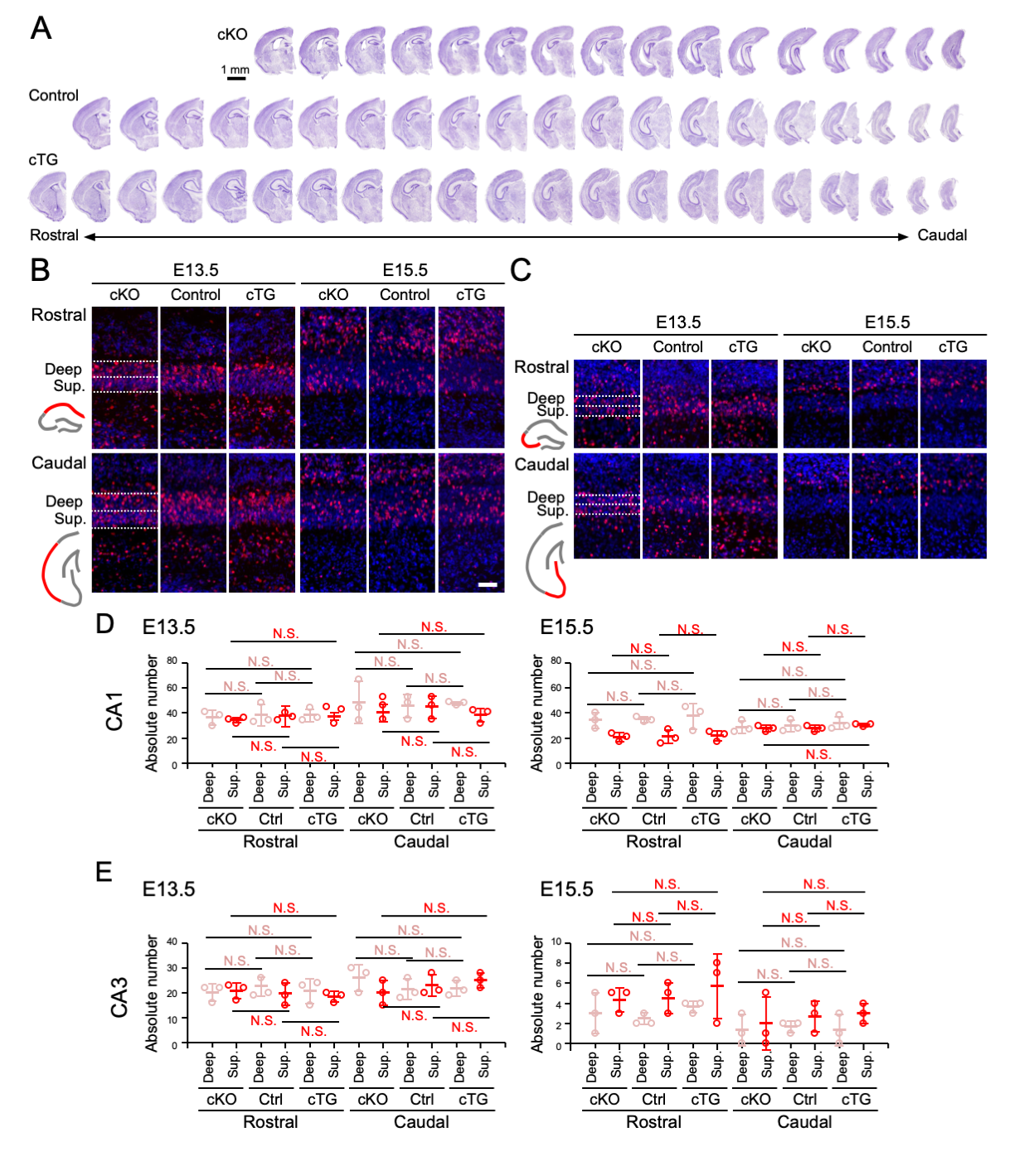

Supplement: S2 Fig — (A) Serial Nissl-stained coronal sections of P7 control, COUP-TFI cTG, and cKO hippocampus along the rostrocaudal axis. (B, C) Distribution of EdU-labeled cells derived at E13.5 and E15.5 in rostral and caudal CA1 (B) and CA3 (C) PCL of COUP-TFI cKO, control and COUP-TFI cTG Hp at P0. (D, E) Quantification of EdU-labeled cell number in the deep and superficial sublayers of CA1 (D) and CA3 (E) regions at the rostral and caudal levels in COUP-TFI cKO, control and COUP-TFI cTG Hp at P0. Scale bars, 1 mm (A); 50 μm (B, C). Statistical analyses were performed using Student t test. The data underlying this figure can be found in S1 Data. (S2_Fig.TIFF) [file pbio.3003355.s002.tiff]

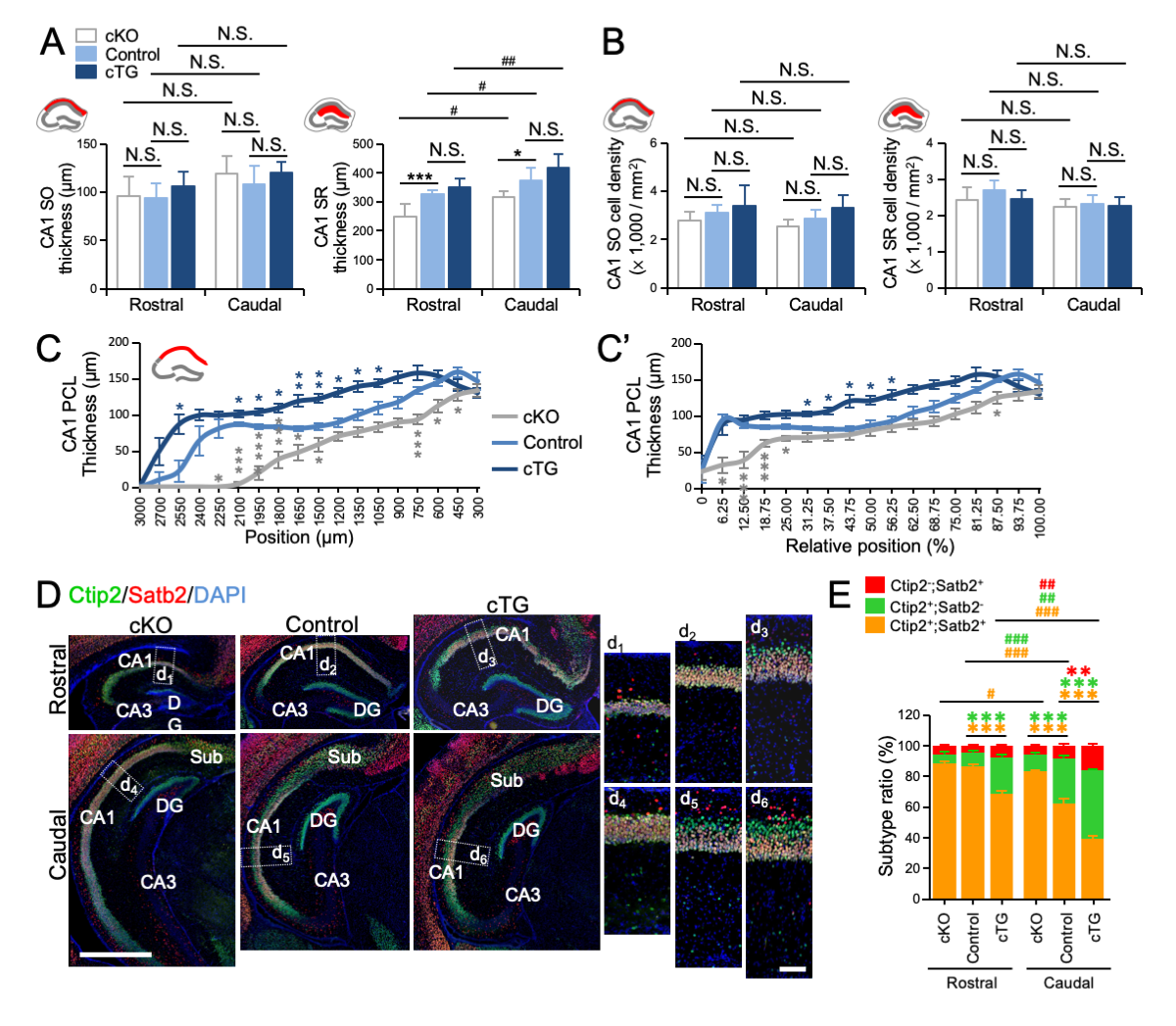

Supplement: S3 Fig — (A, B) Quantification of layer thickness (A) and cell densities (B) in stratum oriens (SO) and stratum radiatum (SR) in COUP-TFI cKO, control, and COUP-TFI cTG Hp at rostral and caudal levels. (C, C’) Morphometric analysis of PCL thickness between COUP-TFI cKO, control and COUP-TFI cTG mice along the rostrocaudal axis. Position number is based on Fig 1D, with normalized positions indicated on the right (C’). (D) Coronal sections of rostral and caudal Hp of P7 COUP-TFI cKO, control and COUP-TFI cTG mice stained with Ctip2 (green) and Satb2 (red). Enlarged images of insets are shown on the right (d1-d6). (E) The distribution of Ctip2- Satb2+ (red), Ctip2+ Satb2- (green), and Ctip2+ Satb2+ (yellow) neuronal populations across the CA1 pyramidal cell layer in COUP-TFI cKO, control, and COUP-TFI cTG hippocampus at the rostral and caudal levels. Scale bars, 1 mm (D); 100 μm (d1-d6). Statistical analyses were performed using Student t test (A, B) and Mann–Whitney U test (C, C’). The data underlying this figure can be found in S1 Data. (S3_Fig.TIFF) [file pbio.3003355.s003.tiff]

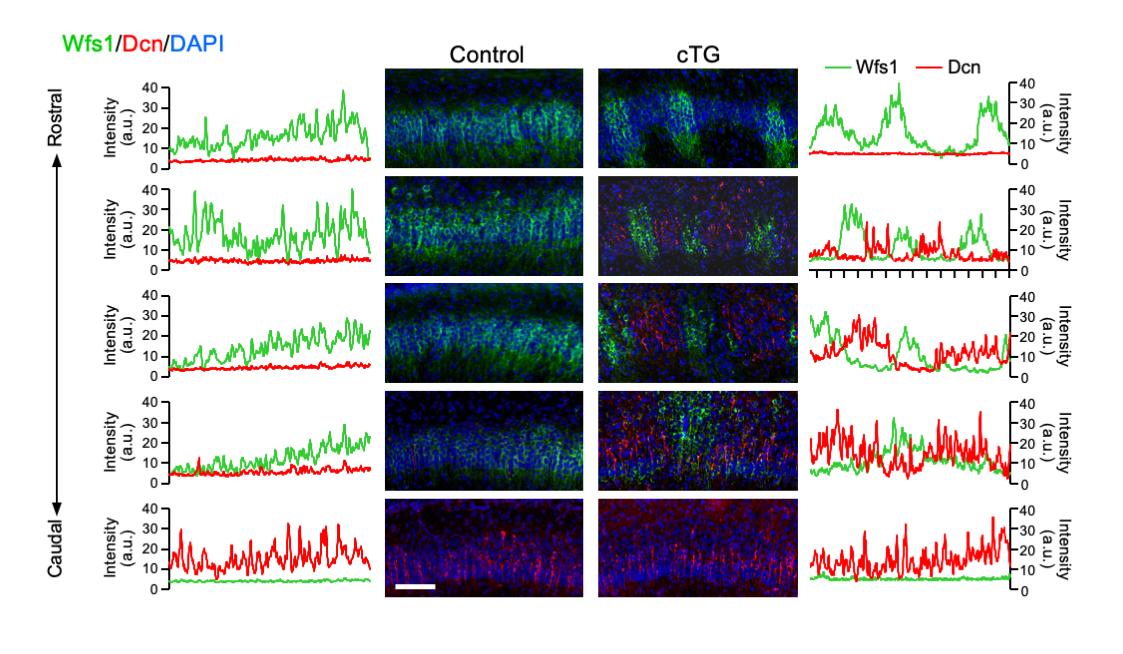

Supplement: S4 Fig — Serial images of immunostaining for Wfs1 (green) and Dcn (red) in coronal sections of P7 control and COUP-TFI cTG CA1, and quantification of signal intensities along the rostral to caudal axis. Neurons in the rostral CA1 were mostly Wfs1+ and those in the caudal CA1 were mostly Dcn+ in control. In COUP-TFI cTG, patches of Wfs1+ and Wfs1- neurons were found in rostral CA1. Dcn+ neurons were ectopically identified in the Wfs1− domain, adjacent to Wfs1+ neurons in rostral CA1 in COUP-TFI cTG. However, the ectopic Dcn+ cells were not present in most examined sections. Scale bars, 100 μm. (S4_Fig.TIF) [file pbio.3003355.s004.tif]

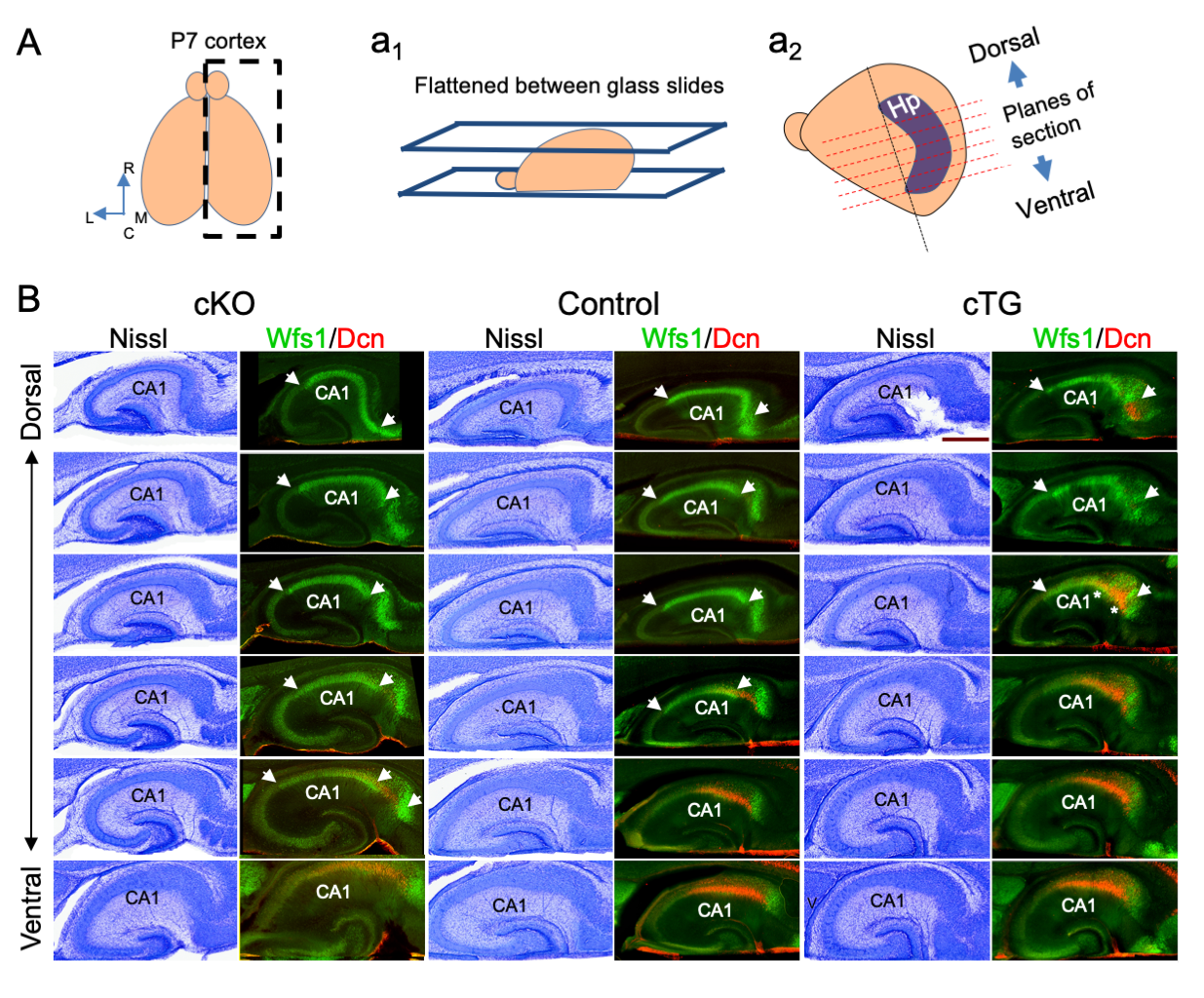

Supplement: S5 Fig — (A) Diagram illustrating the flattening and sectioning. P7 cortical hemispheres were dissected and flattened between two glass slides (a1). Transverse hippocampal sections were obtained by slicing perpendicularly to a line connecting the dorsal and ventral poles (a2). (B) Nissl staining (left) and immunostaining for Wsf1 (green, a dorsal CA1 marker) and Dcn (red, a ventral CA1 marker) in transverse sections of the hippocampus from indicated genotypes. B) Nissl staining (left) and immunostaining for Wsf1 (green, a dorsal CA1 marker) and Dcn (red, a ventral CA1 marker) in transverse sections of the hippocampus from indicated genotypes. The size of Wfs1 expression domains (between arrowheads) was increased in cKO and decreased in cTG. Ectopic Dcn expression was found in the dorsal hippocampus of cTG (*). Scale bar, 500 μm. (S5_Fig.TIFF) [file pbio.3003355.s005.tiff]

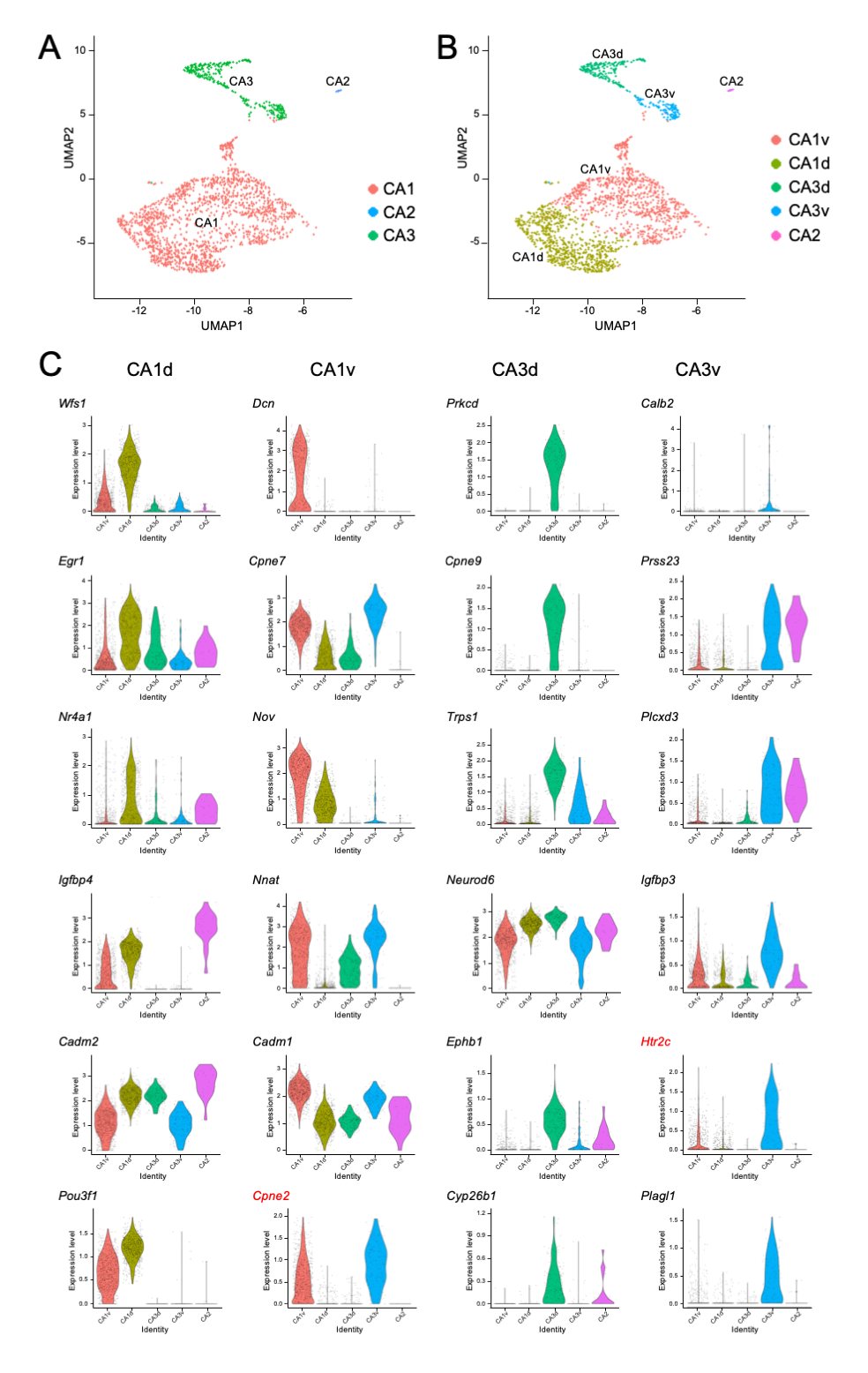

Supplement: S6 Fig — (A) UMAP representation of excitatory neurons from hippocampal CA regions (based on scRNAseq database [30])colored according to CA region. (B) UMAP representations of dorsal and ventral CA1 and CA3 subregions, as well as CA2. (C) Representative genes enriched in the dorsal and ventral CA1 and CA3 regions. The data underlying this figure can be found in S1 Data. (S6_Fig.TIFF) [file pbio.3003355.s006.tiff]

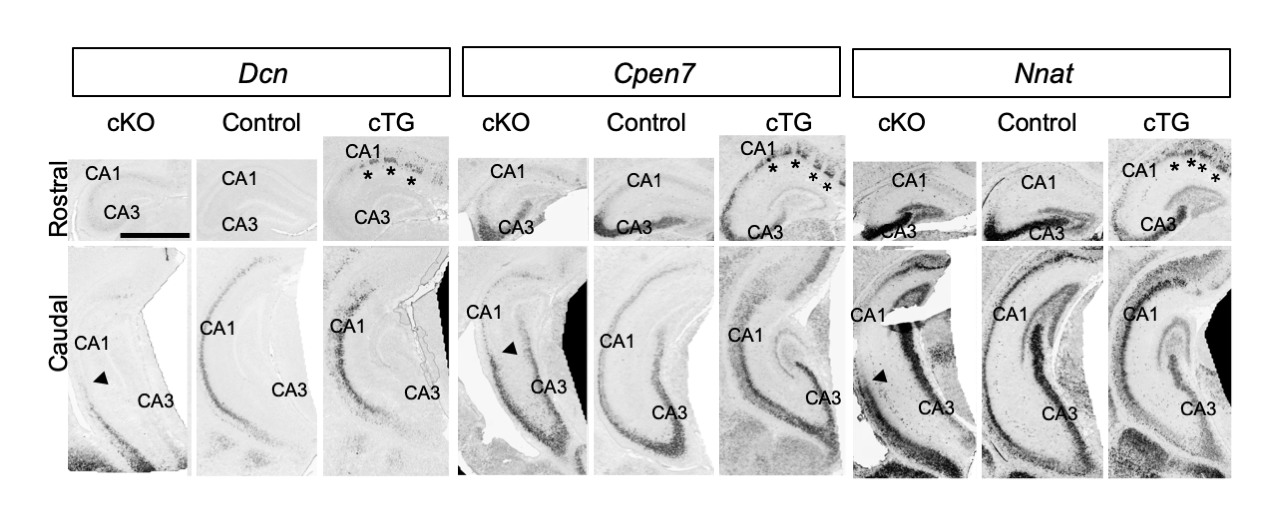

Supplement: S7 Fig — In situ hybridization of Dcn, Cpen7, and Nnat on rostral and caudal coronal sections of P7 control and COUP-TFI mutant Hp. Within CA1, these genes are enriched in the vCA1. The expression of these markers was ventrally shifted in the cKO (arrowheads). Ectopic expression domains were identified in the cTG (marked by asterisks). Scale bar, 500 μm. (S7_Fig.TIFF) [file pbio.3003355.s007.tiff]

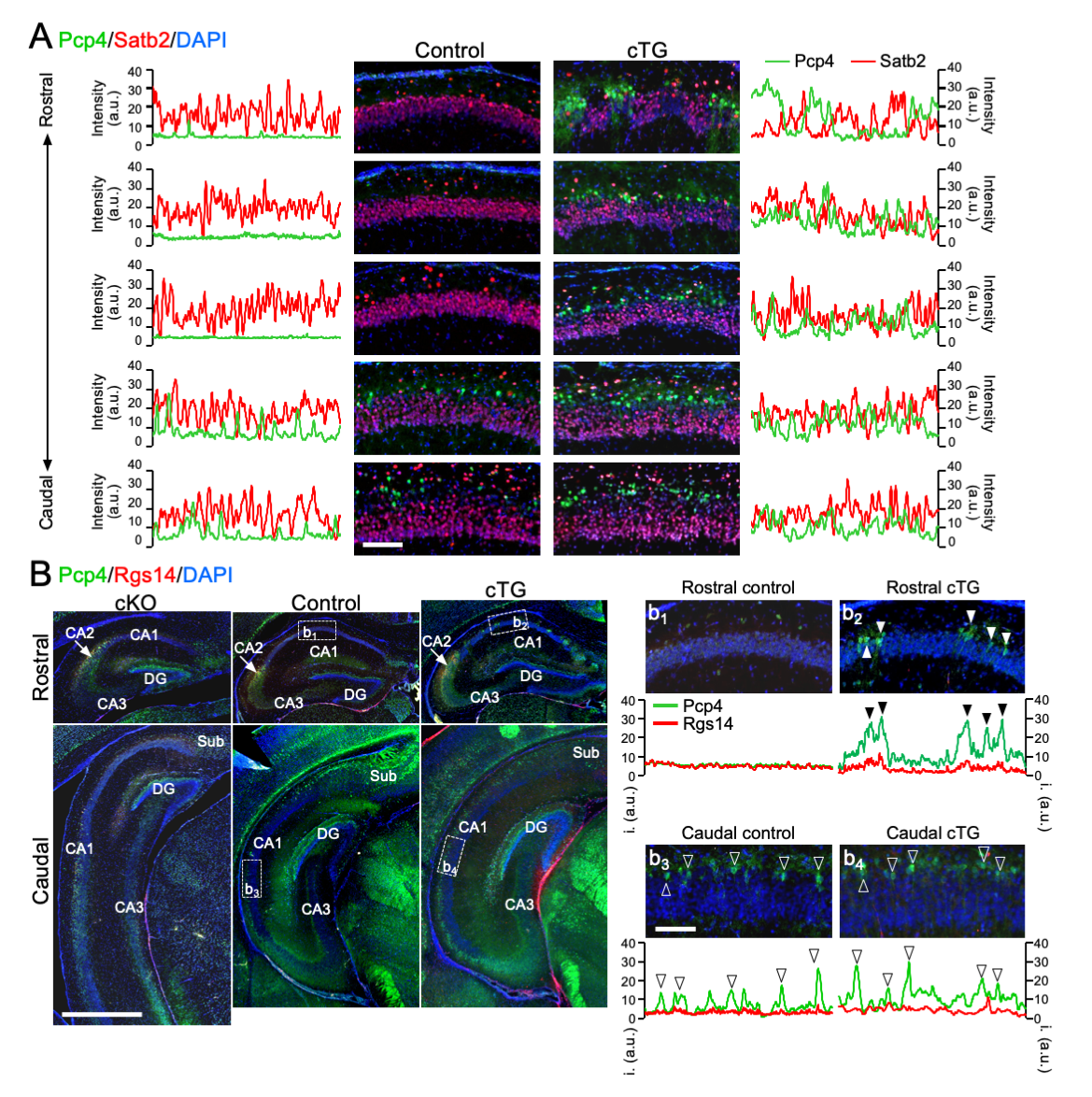

Supplement: S8 Fig — (A) Immunostaining and quantification of signal intensities for Pcp4 (green) and Satb2 (red) in P7 serial coronal sections of control and COUP-TFI cTG hippocampi along the rostral to caudal axis. (B) Immunostaining of Pcp4 and Rgs14 in P7 serial coronal sections of COUP-TFI cKO, control, and COUP-TFI cTG Hp. In the ectopic Pcp4 expression domains within the COUP-TFI cTG CA1 PCL (solid arrowheads), higher Rgs14 expression was detected. However, Rgs14 was not expressed in the Pcp4+ cells in the deep layer of CA1 in the caudal CA1 (open arrowheads). Scale bars, 1 mm (B), 100 μm (A and b1-b4). DG, dentate gyrus; i., intensity; Sub, subiculum. (S8_Fig.TIFF) [file pbio.3003355.s008.tiff]

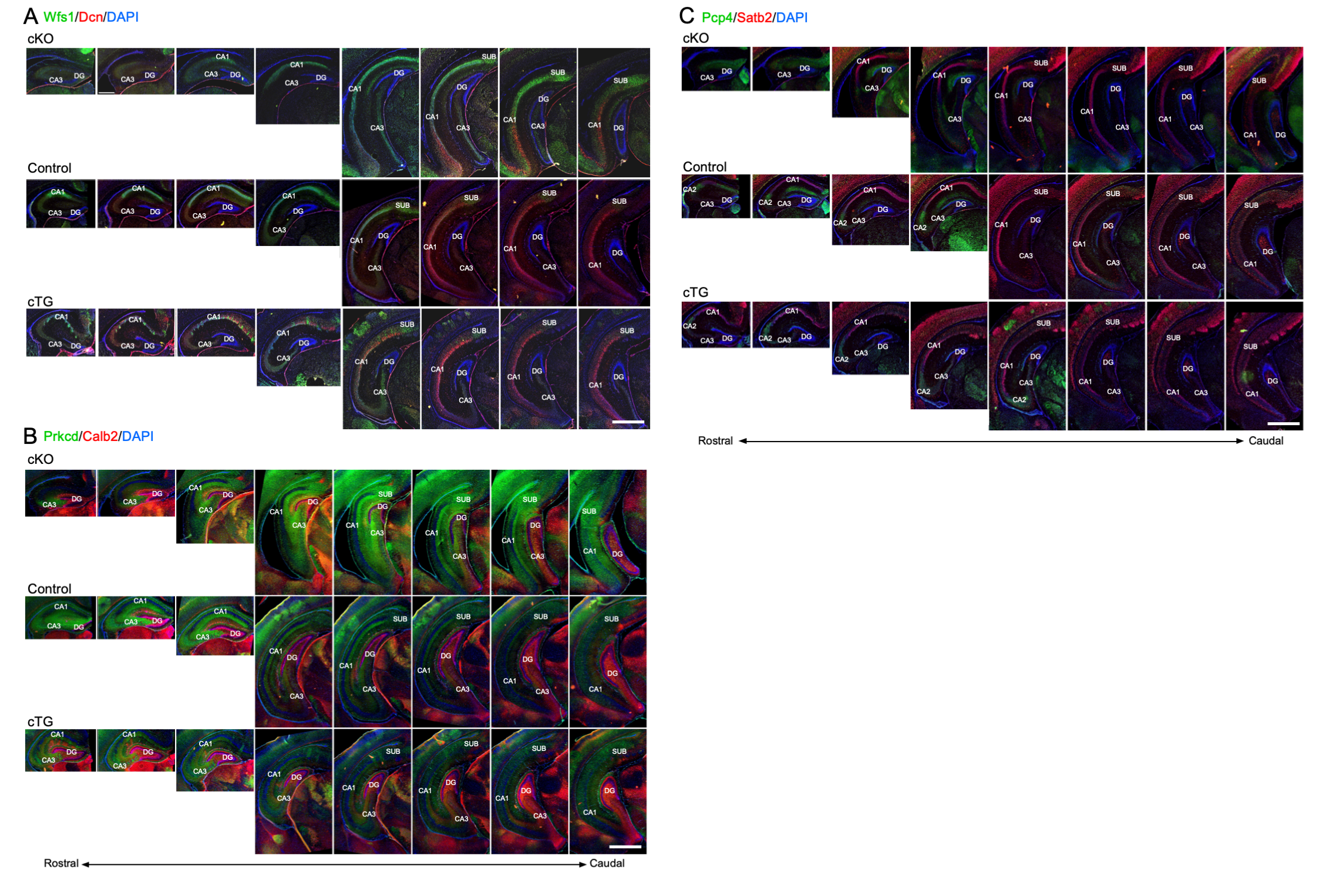

Supplement: S9 Fig — Serial images from immunostaining revealed changes in distinct markers, including Wfs1 (green) and Dcn (red) (A), Prkcd (green) and Calb2 (red) (B), and Pcp4 (green) and Satb2 (red) (C), along the rostrocaudal axis in hippocampi from control and COUP-TFI mutants. Scale bars, 1 mm. (S9_Fig.TIF) [file pbio.3003355.s009.tif]

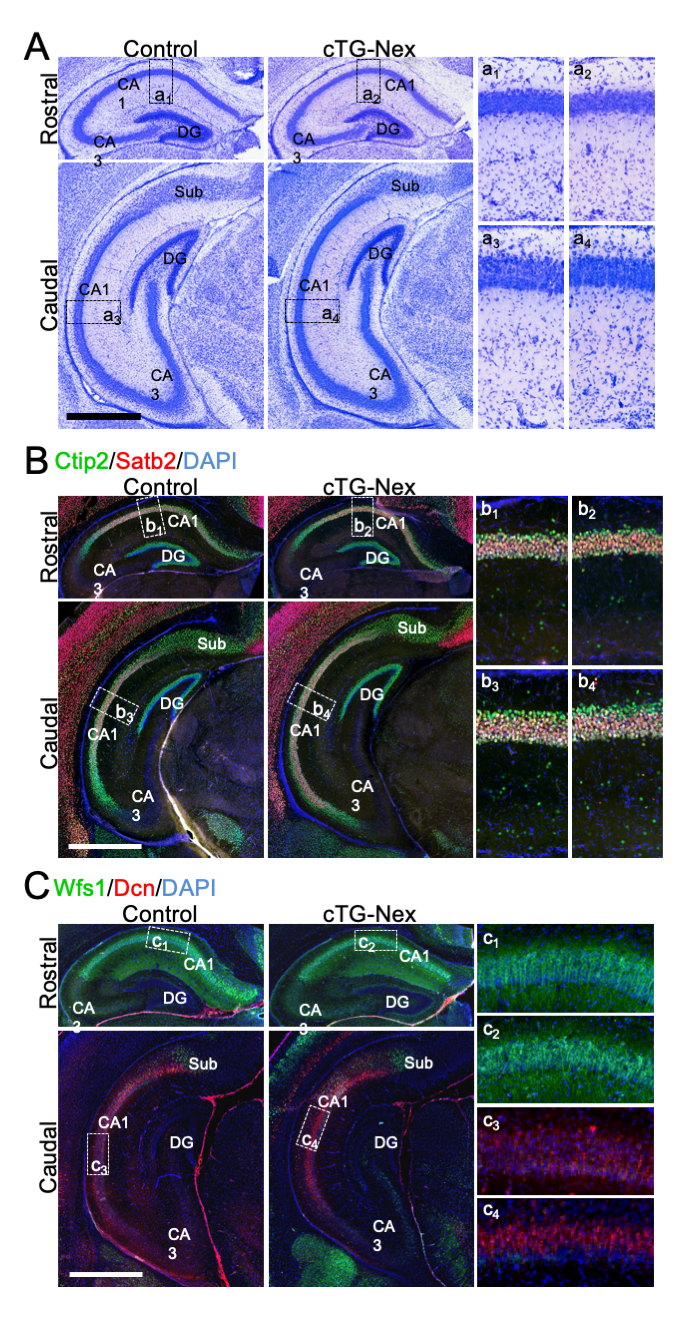

Supplement: S10 Fig — (A) Similar hippocampal structures were detected in Nissl-stained P7 coronal sections of control and COUP-TFI cTG-Nex (COUP-TFITG/O; NexCre/+) cortices at rostral and caudal levels. Enlarged images of insets are shown on the right (a1-a4). (B, C) Immunostaining of Ctip2 (green) and Satb2 (red) (B) and Wfs1 (green) and Dcn (red) (C) in rostral and caudal hippocampi of control and COUP-TFI cTG-Nex revealed similar lamination in both rostral and caudal CA1. Enlarged images of insets are shown on the right (b1-b4, c1-c4). DG, dentate gyrus; Sub, subiculum. Scale bars, 1 mm (A-C),100 μm (a1-c4). (S10_Fig.TIFF) [file pbio.3003355.s010.tiff]
