# Supplementary material for: A developmental gradient of COUP-TFI expression regulates the relative size of hippocampus dorsal and ventral subregions
Source: PLoS Biol. 2025 Aug 25;23(8):e3003355. doi: 10.1371/journal.pbio.3003355 (PMC12396750; doi:10.1371/journal.pbio.3003355)
Supplement: S1 Table — (S1_Table.PDF) [file pbio.3003355.s012.pdf]

S1 Table. Antibody List

Antigen	Company	Cat. No.	Clone	Host	Antibody Dilution	Antigen Retrieval	Major Distribution
COUP-TFI	Perseus Proteomics	PP-H8124-00	H8124	mouse	1:500	v	
Ctip2	Abcam	ab18465	25B6	rat	1:500	v	CA1, DG
Satb2	Abcam	ab92446	EPNCIR130A	rabbit	1:200	v	CA1
Pcp4	Altas Antibodies	HPA005792	polyclonal	rabbit	1:500	v/x	CA2
Rgs14	Antibodies Inc	75-170	N133/21	mouse	1:1000	v	CA2
Dcn	R&D Systems	AF1060	Polyclonal	goat	1:500	v/x	CA1v
Wfs1	Abclonal	A1705	Polyclonal	rabbit	1:500	v/x	CA1d
Prkcd	Abclonal	A7778	ARC1434	rabbit	1:200	x	CA3d
Calb2	Merck Millipore	MAB1568	6B8.2	mouse	1:200	v/x	CA3v, DG

DG, dentate gyrus; v, works with antigen retrieval; x, works without antigen retrieval; v/x, works with or without antigen retrieval.
