# Supplementary material for: A developmental gradient of COUP-TFI expression regulates the relative size of hippocampus dorsal and ventral subregions
Source: PLoS Biol. 2025 Aug 25;23(8):e3003355. doi: 10.1371/journal.pbio.3003355 (PMC12396750; doi:10.1371/journal.pbio.3003355)
Supplement: S2 Table — (S2_Table.PDF) [file pbio.3003355.s013.pdf]

S2 Table. PCR Primer List

Gene/ mRNA target	Forward	Reverse
Wfs1	GATGAAGATGAGGACGAGCT	CCTTGGAGGCTACGTCAATCA
Egr1	AGCGAACAACCCTATGAGCA	ATGGGAGGCAACCGAGTCGTT
Nr4a1	GTGCAGTCTGTGGTGACAAT	GGCAGATGTACTTGGCGCTT
Igfbp4	CGGAGCAAGATGAAGATCGT	GGGATGATGAAGAGGTCTT
Cadm2	TGGCACTTACCGATGTGAAG	CCACTATTCCTCCGATGAGA
Pou3f1	ACAGCCTGCAACTGGAGAA	GCGCATAAACGTCGTCCAT
Rbfox3	ACCACTCTCTTGTCCGTTTG	GGCTGAGCATATCTGTAAGCT
Dcn	ACTCTCCAGGAACTTCGTGT	AGTCCCTGGAAGGCTCCGTT
Cpne7	CACCACATCAACCCTTACCA	CAGAGCAGAAAACCGCTTGT
Nov	TCGCCAGTGTGAGATGGTAA	GTGGATGGCTTTCAGGGATT
Nnat	TGGTGGAGGAAGAGGGTTAA	CACATTTTGGGGAGGGCTTT
Cadm1	ACTTCTGCCAGCTCTACAC	CCTTCAACTGCCGTGTCTTT
Cpne2	GTGTGGAAACCATTCACTGT	CAGCACAGAGGTCTGGAA
Prkcd	TCTGCGGCACTCCTGACTAC	CTGGTTGTGAACTCGCCAAT
Cpne9	CACTGTGCAACGGAGACTAT	CTGGTTGTGAACTCGCCAAT
Trps1	AGGTCAACCGTTCTGTGCTT	TGTTGCCTTGGCAATCTGGA
Neurod6	TCTGAGGATTGGCAAGAGAC	TGGCGTTGAGCTGTAAGCA
Ephb1	CAGGTCAGTGCCACCATGA	GATACGTGCTGTGTTGGTCT
Cyp26b1	GAGAGCAGCAAGGAACATG	CAAGGATGTGCTTGCACTG
Calb2	CCTGAAGGATCTGTATGAGA	AGCACAATCTCCAGGTCCTT
Plcxd3	CCCTCACCAATTTAGCCAT	GCATCAACTTCTTGGCTACA
Prss23	TGAGGATGTGGAAGAGACCA	AGGCGTGATTCTAACTGCCA
Igfbp3	GTTCCTCAATGTGCTGAGT	TGTCCACACACCAGCAGAA
Htr2c	CGGTTCAATTCGCGGACTAA	GTCATTGAGCACGCAGGTA
Plagl1	CAAGTGCTCGAAGGCTGAGT	GTGAGTACACTGGTGAATCTCT
