# Supplementary material for: A developmental gradient of COUP-TFI expression regulates the relative size of hippocampus dorsal and ventral subregions
Source: PLoS Biol. 2025 Aug 25;23(8):e3003355. doi: 10.1371/journal.pbio.3003355 (PMC12396750; doi:10.1371/journal.pbio.3003355)
Supplement: S3 Table — (S3_Table.PDF) [file pbio.3003355.s014.pdf]

S3 Table. ISH Primer List

Gene	Forward	Reverse
COUP-TFI	AGGCCAGTATGCACTCACAAAC	AGTCTCTAGGGAGTCAGGGAGC
Lef1	AGAGAACACCCTGATGAAGGAA	CTTCCTCTTCTTCTTCTTGCCA
Axin2	CCTTGCCAAAACGGAATG	TTTCGTGGCTGTTGCGTA
Wnt3a	GTTCCTACTTGGAGGGGTCTCT	CTATCATACGAGGCTGTCATGC
DCN	CTTCCTTCTGGCACAAGTCTCT	TGTTGTTGTCCAAGTGGAGTTC
Cpen7	CAGTGGCTACAGGTGGACAGA	ACCCTGTGGAGCTTCAGGTCT
Nnat	CTTCTCGACCAGCATGGG	TTGACCACAACTGCTGCG
