# Supplementary material for: A developmental gradient of COUP-TFI expression regulates the relative size of hippocampus dorsal and ventral subregions
Source: PLoS Biol. 2025 Aug 25;23(8):e3003355. doi: 10.1371/journal.pbio.3003355 (PMC12396750; doi:10.1371/journal.pbio.3003355)
Supplement: Attachment — Submitted filename: Reviewer responses-0611.pdf [file pbio.3003355.s018.pdf]

We appreciate the enthusiasm of the reviewers for our study. Based on the reviewers' suggestions, we have modified the manuscript. Here we provide a point-by-point response to comments of each reviewer. The Reviewers' comments are in blue italic and our responses follow in plain text. We quote and address all comments made by the reviewers.

Reviewer #1: Tseng et al. work focuses on understanding the role of COUP-TF1, a transcription factors, in the development of the hippocampus, particularly, in the patterning of hippocampal subregions. The authors use a combination of anatomical and molecular approaches to analyze how a deficit (cKO) or an overexpression (cTG) of COUP-TF1 affects the sizes of the different subregions.
The work is mainly descriptive (which I don't see as a negative point), and the authors have carefully analyzed the changes in the cKO and cTG with different markers and across the whole dorso-ventral axes. I particularly appreciate having supplementary figures with additional images to show stainings at different rostro-caudal levels. I feel few points could be better investigated or discussed, but I overall think this work will be rather interesting for the field of developmental neuroscience and those studying the hippocampal regions.

OUR RESPONSE
We thank the reviewer for finding our study interesting.

Major comments:
* *The authors show how changes in the level of COUP-TF1 often leads to the expression of region-specific markers in ectopic regions. For example, the expression of Dcn in patches of CA1 or of Pcp4 in deep PCL in caudal CA1. This is very interesting, though it is not very much discussed in the manuscript. What could be the reason of these ectopic expression? In particular, in the Discussion, the authors mention that their data support the notion that CA1 PC and CA3 PC derive from different progenitors, but vCA1 and dCA1 would derive from the same progenitors. If that is the case, how do we explain the ectopic cells? Are those the consequence of migration defects (like abnormal cells migrating in the wrong place). Or are they the product of misspecification of specific cells from some specific progenitors? I would appreciate if the authors could speculate in the discussion, as these data might be difficult to interpret for scientists outside of the field.*

OUR RESPONSE
We thank the reviewer for the suggestion. We added the following section to the Discussion (pg.14-15) to better explain the phenotype:
"Interestingly, when COUP-TFI expression was elevated in cTG mice, we observed the formation of ectopic vCA1 and CA2 domains within the dCA1, as well as ectopic vCA3 within the dCA3. This finding is consistent with our previous study, in which COUP-TFI overexpression led to the emergence of ectopic medial entorhinal cortex (MEC) domains in the neocortex (NC) [24]. In that study, we showed that cortical progenitors are multipotent and can give rise to either MEC or NC neurons. The fate choice of these progenitors was determined by two patterning TFs with opposing expression gradients: high level of COUP-TFI (expressed in a high-caudal-to-low-rostral expression gradient in the cortical progenitors) promoted MEC cell fate, while another, yet undefined TF (expressed in a high -rostral-to-low-caudal gradient) likely promoted NC cell fate. In our cTG model, COUP-TFI overexpression created a condition in which both patterning TFs were expressed at high levels, resulting in a zone of fate instability where progenitors could adopt either fate. Subsequently, the physical segregation of these distinct cell populations was driven by differential cell affinities.

In the current study, we propose that a similar mechanism governs the Hp dorsal-ventral patterning. COUP-TFI, which is normally expressed in a high-caudal to low-rostral gradient in HNE, appears to promote ventral hippocampal identity. Its ectopic overexpression in the rostral HNE, which normally gives rise to dorsal Hp, generates an instability zone where both dorsal and ventral Hp neuronal types are produced. These dHp and vHP neurons then undergo segregation, likely via differential cell adhesion, leading to the formation of ectopic ventral domains within dorsal hippocampal regions. Overall, our findings suggest that distinct hippocampal subregions, including dorsal and ventral CA1 and CA3, are likely segregated by

differential cell adhesion affinities, and COUP-TFI plays a key role in patterning of these subregions."

* *The authors have some data with Edu labeling in the supplementary figures, where they show that there is no increase in cell production. These data nicely fit with what is reported in Fig.2B. However, I am sure that the authors known that the literature has showed a difference in the DOB of deep VS superficial and that CA3 and CA1 cells follow different timeline for DOB and peak of neurogenesis. It would be extremely interesting if the authors could add i) analyses of the deep VS superficial part of the PCL (a simple 50-50 division could work, or authors could use speific markers to distinguish them); and ii) also similarly analyses of CA3. This would help in establishing if the differences observed are also caused by a mis-timed differentiation or neurogenesis.*

OUR RESPONSE

We thank the reviewer for the helpful suggestion. We have now further quantified the number of EdU labeled cell in deep versus superficial parts of the PCL in both CA1 and CA3 across all genotypes (Figures S2B-2E). Our analysis revealed no significant differences in the temporal patterns of neurogenesis in control and COUP-TFI mutant Hp.

* *Fig. 4B. The high-caudal-low-rostral gradients are a bit hard to catch, as they seem a bit subtle. In particular, it almost seems like it is more the size of the HNE that changes, rather than the signal intensity (in particular for Axin2). Perhaps the authors could use some dashed lines to show the areas we should focus on and have some higher magnification images that better support their claim.*

OUR RESPONSE

We thank the reviewer for this great suggestion. We have now added dashed lines in Figures 4B and 4C to indicate the HNE and cortical hem (CH), which helps to better visualize and appreciate the gene expression gradients.

Minor comments:

* *From Fig.2A-2c, it is unclear what is counted in the B and C. The text seems to suggest all the CA1 area, but the figure and legend say "CA1 PCL". The authors should specify. And since it seems that also the neuropil is affected in the cKO and the cTG, the authors might consider analyzing those differences and discuss what could be the reason, in case they have analyzed only the PCL.*

OUR RESPONSE

We have clarified this point in both the main text and figure legends to indicate that we were measuring the thickness and cell density of the CA1 PCL. In addition, we have now included new analyses of the stratum oriens (SO) and stratum radiatum (SR), presented in Figures S2F and S2G. These results show that the major structural differences observed in COUP-TFI mutants are localized to the PCL.

* *It is rather curious that the authors never analyze the Dentate Gyrus. Since the paper is about subregions of the hippocampus, there could be an expectation that this subregion is also included in the analysis. While I am not asking the authors to add work on the DG, perhaps they should specify in the text (and perhaps the title) that they are analyzing only the CA regions, which could be called "Hippocampus proper".*

OUR RESPONSE

We thank the reviewer for the insightful suggestion. In response, we have revised the title of our paper to: …"Subregions of the Hippocampus Proper"…
As noted, our current study does not include analysis of the dentate gyrus (DG). However, the DG is indeed known to exhibit dorsoventral differences in both axonal projections and neurogenesis. In future work, we plan to investigate whether the DG can also be molecularly subdivided into dorsal and ventral compartments, and whether COUP-TFI plays a role in DG patterning and possibly in regulating DG neurogenesis.

* *For the experiments in Fig.S2, do authors count only cells in the PCL? If so, they should specify it.*

OUR RESPONSE
Yes, we only measured the PCL, which is now clarified in both the main text and figure legends. In addition, we have now included the cell counts for the stratum oriens (SO) and stratum radiatum (SR) in new Fig S2F and S2G.

* *Related to the Edu experiments, they seem to be completely missing from the Methods section. Authors should add that.*
OUR RESPONSE
We thank the reviewer for pointing out this oversight. We have now included the missing information in the Methods section.

* *Fig.S4, legend: typo in Reference for RNA-seq database (I guess it's #30 and not #3).*
OUR RESPONSE
We thank the reviewer for noticing this error. We have updated the reference.

* *Fig.4D, authors should explain somewhere how the quantification was done and how the "square" was obtained.*
OUR RESPONSE
We now included a new paragraph in the Methods section (pg. 21): "To determine gene expression gradient (Fig 4D), HNE was divided into six equally spaced domains on five adjacent coronal sections. The average intensity of RNA in situ hybridization color reaction signals was measured within each domain. These intensity values were normalized to the background level of each section. Relative expression levels were then calculated by comparing the average intensity of each domain to the highest expression observed, which was in the most caudal ventral domain. The average intensity values of each domain was then visualized as a heatmap to illustrate the gradient."

* *The methods section in general is lacking a bit in details regarding equipment models and parameters used. For example, nothing about that is reported for the Light-Sheet microscope. It would be nice if the authors could add that and re-check their methods section.*
OUR RESPONSE
We have updated the methods section and now included the equipment models and parameters.

* *Fig.2. In D, an explanation of the arrow heads is missing. In G, I assume V/D cell ratio means the authors first did the Dcn/Wfs1 ratio for the Ventral and Dorsal region and then calculated that ratio. That could be explained a bit better in the legend.*
OUR RESPONSE
We have revised it to the ration of " # of Dcn cells/ the # of Wfs1 cells" in the figure to improve clarity.

* *I couldn't find the Supplementary Movie 1 in my files.*
OUR RESPONSE
We have included the supplementary movie.

Reviewer #2: *Outstanding and novel results on the role of Nr2f1 in regulating regionalization of the mouse hippocampus studied using Nr2f1 loss and gain of function mutants. The figures are excellent. Quantifications are carefully done. The writing is precise.*

Therefore, the paper Merits publication with some minor editing and no further review.
OUR RESPONSE
We thank the reviewer for the very positive comments.

For the Intro and/or Discussion, please reference Faedo et al, Cerebral Cortex, 2007 and Ypsilanti et al PNAS 2021. These papers showed that in the cerebral cortex, Nr2f1 alone or with Nr2f2, promote ventral identity (see Figure 3 of PNAS paper). Figure 8 of cerebral cortex paper showed that loss and gain of Nr2f1 regulates D/V patterning of the cortex. While it is well known that Nr2f1 promotes caudal identity, it is not well appreciated that it also has a key role in patterning the ventral cortex. This is highly relevant to Nr2f1's role in hippocampal D/V patterning.

Regarding the NR2F1 ChiP-seq - please reference Ypsilanti et al PNAS 2021 who also performed this type of analysis on the E12.5 cortex, although they focused on the neocortex and Nr2f1 binding to regulatory elements of other TFs. They did report that Nr2f1, Pax6, Emx2 Lhx2 and Pbx1 showed co-binding to regulatory elements near several WNT receptors (Frz1,5,7 & 8; Supplemental Figure 2 I).

Regarding WNT signaling, Faedo et al, Cerebral Cortex, 2007 provided evidence that loss of Nr2f1 increased the ventral spread of the bat-gal WNT reporter allele, whereas gain of Nr2f1 repressed Bat-gal activity.

OUR RESPONSE
We thank the reviewers for the suggestions regarding relevant references. We have incorporated these citations into the revised manuscript.

Reviewer #3: Tseng et al present a nice body of work examining the role of COUP-TF1 in patterning the dorsal-ventral axis of the hippocampus. Their work adds to the literature by using both knock out and an enhanced expression of COUP-TF1, and they conclude that COUP-TF1 knock out leads to the expansion of the dorsal hippocampus (while reducing the ventral hippocampus) and that overexpression has the opposite effects (reduced dorsal hippocampus, expanded ventral hippocampus with ectopically expressed ventral hippocampal cells). I greatly appreciate their attention the different subregions of the hippocampus.

OUR RESPONSE
We thank the reviewer for appreciating our study.

Because the curvature of the hippocampus can make it difficult to assess and align sections of a shortened or elongated hippocampus with that of wild-type mice, the analyses with Nissl-stained sections are harder to believe than the tissues that are stained with specific markers of cell type and location along the dorsal-ventral axis. In particular, the ventral morphology of the cKO is quite challenging to align with the other two genotypes and it is difficult to tell how much of that is due to cutting this more caudal tissue at a very different angle due to a shortened curvature. Perhaps a more appropriate prep would be to dissect the hippocampus and section the flattened hippocampus to compare dorsal, intermediate, and ventral across genotypes.

OUR RESPONSE
We thank the reviewer for the insightful comments. We are aware that the curvature of the hippocampus can complicate the alignment and comparison of sections across different genotypes. To address this challenge, we carefully compared hippocampal phenotypes between control and COUP-TFI mutant mice using whole-brain imaging (Figure 1C), and by analyzing multiple coronal sections along the rostral–caudal axis throughout the entire hippocampus (Nissl staining in Figure 1D; multiple molecular markers in Figure 2, 3 and summarized in S9). We believe that our comprehensive approach enabled us to detect patterning changes in COUP-TFI mutants that were missed by previous studies.

In response to the reviewer's suggestion, we also flattened the cortex and sectioned the hippocampus perpendicular to its D-V axis as shown in Figure S5. We found consistent patterning changes in COUP-TFI mutants.

Similarly, scaling up the cKO and scaling down the cTG is also difficult to interpret because it isn't clear if the differences in sizes are evenly distributed, or how they relate to the control. Instead, it might be useful to do percent of the axis, and certainly the qRT-PCR analysis of the whole hippocampus is more compelling.

OUR RESPONSE
We thank the reviewer for the helpful suggestion. We now use "percent of the axis" in Figures 2F and 3D to assess patterning shifts within the hippocampus across different genotypes. We carefully aligned the hippocampal sections from different genotypes, based on their relative position in the entire hippocampus along the dorsoventral axis. We also appreciate the reviewer's support of our use of qRT-PCR to evaluate these patterning changes. Additional results for genes enriched in either dorsal or ventral hippocampal regions have been included and are presented in Figures 2H and 3B.

Of note, the labeling of vCA1 in the cKO image of Figure 2A is questionable. The authors have identified a window of very thin pyramidal layer to measure and claim is thinned in the cKO mouse, but they have labeled the CA1 layer quite extensively into what is likely the CA3 layer, based on the widening of the cells. Clearer delineations of the subfields would give the reader more confidence in the assessment of the subregions by this group.

OUR RESPONSE
We thank the reviewer for this comment. We have corrected the labeling accordingly. Additionally, we performed immunostaining for Satb2 and Ctip2 to carefully define CA1 across different genotypes (new Fig S3D).

I would like to note a few issues in the discussion:
"In this study, we showed that even at an early postnatal stage (P7), when not all the subregion-specific genes are fully expressed, the Hp can still be divided into dorsal and ventral compartments using a handful of marker genes. This suggests that Hp compartmentalization takes place early in development, well before the Hp begins to show adult-like functions at three to four weeks after birth [39]."

While this is true, they do not note that both connectivity of the hippocampus with its main cortical input the entorhinal cortex, as well as dorsally or ventrally enriched gene markers at the dorsal/ventral extremes of the neonatal hippocampus are identifiable at birth (O'Reilly et al Brain Structure and Function 2015).

Additionally, they state: "Further compartmentalization and refinement of these subregions likely takes place during Hp maturation, as physical activities are known to significantly contribute to hippocampal development in humans [40, 41]."

Isn't "further compartmentalization and refinement" the definition of maturation? This sentence feels empty and vague and the references 40 and 41 are taxi driver studies; are these two studies really support for the role of "physical activities to significantly contribute to hippocampal development in humans?" Instead the authors could cite articles that indicate the sensory input is relevant for hippocampus maturation, such as studies of early eyelid opening leading to earlier maturation of the dentate gyrus (e.g. Dumas Dev Psychobiology 2004), or perhaps relate the timing of their studies to other changes in maturation across sensory systems, because sensory input is important for hippocampal-based learning an memory.

OUR RESPONSE

We thank the reviewer's insightful comments. We have revised this part of the discussion accordingly and included the appropriate citations.

Finally, they state that the hippocampus has been functionally described to have gradients along the axis but that "when analyzed at the molecular level, the hippocampus can be divided into distinct domains. Different sets of genes are enriched in specific subregions of the adult Hp, including the dHp, vHp, and an intermediate region between them [10, 38]."

Citation 38 by Fanselow presented the case that the two domains may not be distinct, based on gradients of molecular labeling. If you look at the extremes, you can identify distinctions, but the boundaries are not necessarily clear cut.

OUR RESPONSE

We agree with the reviewer and now have revised the text as follows: "Gene expression gradients are also present in the Hp along the dorsoventral axis. Additionally, different sets of genes have been shown to be enriched in specific subregions of the adult Hp, including the dHp, vHp, and an intermediate region between them, although the precise boundaries of these subregions have not been clearly defined (Dong et al., 2009; Fanselow and Dong, 2010)."

A few minor comments:

1) The use of dHp and vHp and then swapping back and forth between this nomenclature and the rostral-caudal nomenclature makes the manuscript harder to follow.

OUR RESPONSE

We mostly use rostral caudal to describe anatomical location. In the case of the cKO, the Hp with dorsal identity might be located in a more caudal location compared to the control. We did our best to use the nomenclature consistently.

2) in Figure 2, it is very hard to see the letters a1-a6 on the images.

OUR RESPONSE

We thank the reviewer's suggestion. We have made the changes to Figure 2 accordingly.

Reviewer #4:

In their manuscript entitled "Determining the relative sizes of hippocampal subregions along the dorsal-ventral axis by COUP-TFI expression gradient", Tseng and colleagues highlight the role of transcriptional regulator Couptf1 in the regionalization of the hippocampus and investigate how the expression gradient hippocampal progenitors (high-caudal to low-rostral) may contribute to distinguishing the fate of dorsal and ventral hippocampal regions. The authors used Couptf1 conditional knockout (cKO) and Coutf1 overexpression (cTG) models to study the effects of decreased or increased Couptf1 expression in the emergence of dorsal and ventral hippocampal fate and in the molecular regionalization of the hippocampus.

Using anatomical analysis and expression of molecular markers they proposed that Couptf1 deletion results in the expansion of the dorsal hippocampus at the expense of the ventral region, and that increased Couptf1 expression results in ectopic generation of ventral hippocampus in the dorsal region. Moreover, they point to Wnt signaling as a mechanism regulated by Couptf1 to control dorsoventral regionalization in the hippocampus.

Overall, Tseng et al. highlight an important concept linking Couptf1 to the regionalization of the hippocampus. The involvement of Couptf1 in hippocampal regionalization has been proposed previously, and this study supports Couptf1's role in hippocampal morphogenesis. This study offers a new perspective on how Couptf1 may regulate hippocampal regionalization, contrasting the current view in the literature. However, the study presents important shortcomings in supporting their main claims.

OUR RESPONSE

We thank the reviewer for finding our study important and for acknowledging that it offers a new perspective on hippocampal regionalization.

Main concerns:
1. One of the author's main claims is that Couptf1 regulates the dorso-ventral balance of CA1, as well as the position of CA2. The authors claim that the dorsal hippocampus (dHP) expands at the expense of the ventral hippocampus (vHP) in the absence of Couptf1. Previous publications (cited by the authors) using two different Couptf1 mutant models (including the Emx-Cre;Couptf1fl/fl system used in this study) have shown that Couptf1 is necessary for the specification and differentiation of the dorsal hippocampus, and its mutation mostly affects the differentiation of the dorsal CA1. This is in stark contrast with the author's main claims however the authors do not discuss this discrepancy.

The authors show that the HP is smaller in the Couptf1 cKO (as observed in previous studies), and that the thickness of the pyramidal layers in CA1 and CA3 changes. These morphological changes indicate the abnormal development of these fields. The study argues that the reduced thickness and increased cell density in the cKO CA1 and the increased thickness and decreased cell density in the cTG CA1 suggested that the Hp was "dorsalized" in the cKO and "ventralized" in cTG. These anatomical changes are unlikely to reliably distinguish regional differences given the dysmorphism of the HP in the cKO.

OUR RESPONSE
We agree with the reviewer that we cannot distinguish the regional difference solely based on the anatomical changes. This is the reason that we used additional region-specific markers to demonstrate the patterning changes in the COUP-TFI mutants (Figures 2, 3, S3-5, S7 and S9).

Their claim that dHP expands at the expense of the vHP is mostly supported by the expanded expression of the Wsf1 marker in CA1 from rostral to caudal (Figure 2D). Of note, expression of Wsf1 in dHP CA1was shown in the study of Yang and colleagues (doi: 10.7554/eLife.86940) in the same Couptf1 cKO model (Emx1Cre; Couptf1 fl/fl), and appeared to be reduced rather than expanded. There is no discussion regarding the choice of the markers, and given the discrepancy with the literature, it diminished the strength of the data yielded by markers that may not be reproducible across studies.

OUR RESPONSE
The reviewer is correct in noting that our conclusions differ from those of previous studies (Flore et al., 2017 and Yang et al., 2023). We appreciate the reviewer's careful evaluation of these literatures. Here, we would like to highlight the key differences between our interpretation and those of earlier reports, and to address specific limitations of those two previous studies.
We address the discrepancies on two levels:

I. <u>Analyzing results based on limited number of sections likely leads to misinterpretation</u>: All our studies (Flore, Yang, and ours), agree on an overall reduction in hippocampal size in the cKO animals. This smaller hippocampus is likely to affect the relative location and structure of hippocampal domains on coronal sections—for example, the hippocampus might appear smaller in the rostral sections, where dorsal hippocampus is normally located in the control animals. Yang et al., concluded a diminished dCA1 in the COUP-TFI cKO based on reduced Wfs1 and Ctip2 expression (Figure 2—figure supplement 1 in Yang et al., 2023). In the following figure, we put this data next to ours Figure S9A. At one specific level, we also observed the diminished Wfs1 expression (red box, diminished Wfs1 expression domain pointed by arrows). However, Wfs1 expression varies significantly along the rostral-caudal axis in both control and cKO hippocampi. Although there are levels where Wfs1 expression domain diminished in cKO (pink and red boxes), there are sections where Wfs1 expression is comparable between control and cKO (yellow box). Importantly, we found Wfs1 expression is sustained in the caudal CA1 in the cKO (green box). This suggests the Wfs1-expressing dCA1 is expanded caudally in the cKO. Therefore, we argue that it is inaccurate to conclude a diminished dCA1 based solely on a single or limited number of sections.

II. Relative regional proportions in the context of entire hippocampus: Our analyses did not just focused on the absolute size or location of dorsal and ventral hippocampus. Instead, we aimed to address the dorsal-ventral patterning event in hippocampus, focusing on the relative proportion of dorsal to ventral subregions within the entire hippocampus. As shown in Figures 3D and S9, our analyses revealed a significant increase in the relative size of dHp and a decrease in vHP in the cKO.

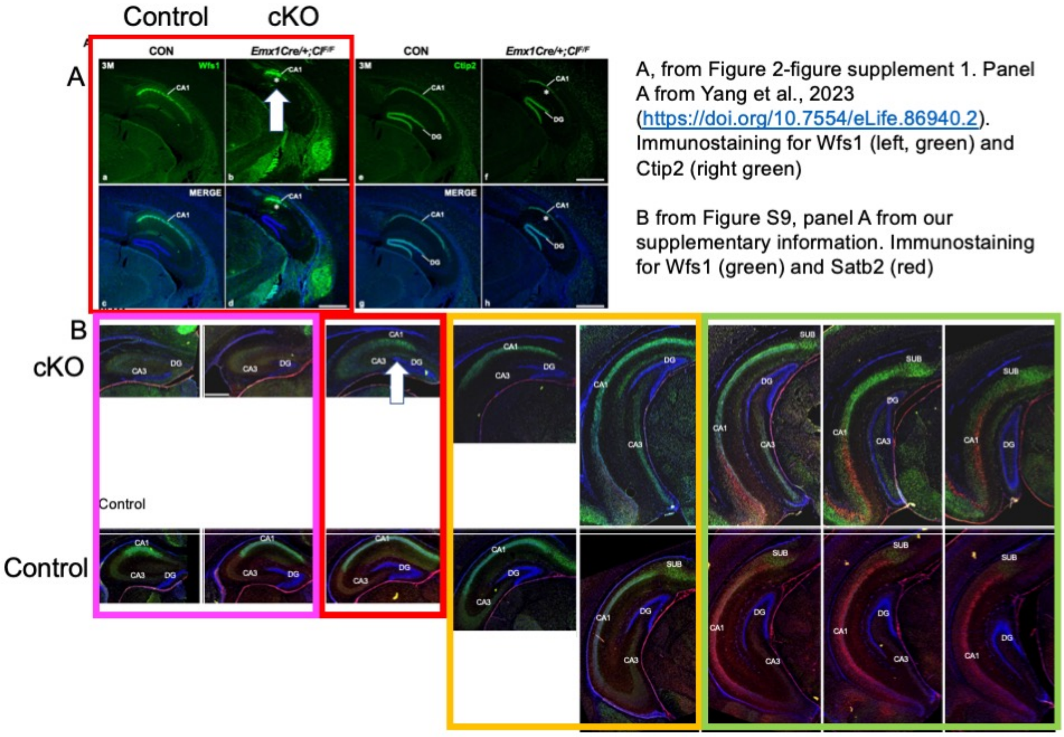

A, from Figure 2-figure supplement 1. Panel A from Yang et al., 2023 (https://doi.org/10.7554/eLife.86940.2). Immunostaining for Wfs1 (left, green) and Ctip2 (right green)

B from Figure S9, panel A from our supplementary information. Immunostaining for Wfs1 (green) and Satb2 (red)

In conclusion, our study demonstrated that "Couptf1 regulates the dorso-ventral balance of CA1". Importantly, we did not draw our conclusions solely from the location of dHp, as done in previous studies, but from comprehensive analyses of the expression of multiple dorsal- and ventral-enriched marker genes across the entire hippocampal axis. Our findings are consistent with prior results, but we interpreted the results in the context of whole-hippocampus patterning rather than on isolated sections.

We have emphasized this point in both the introduction, "However, to our surprise, different from what was reported based on marker gene analyses done on limited numbers of sections, our systematic examination of multiple subregion-specific markers across the entire hippocampus at postnatal day 7 (P7) revealed that in the COUP-TFI conditional knockout (cKO) mice, where COUP-TFI is deleted in the cortex by the Emx1-Cre, the relative size of dHp was actually expanded at the expense of the vHp." (pg. 4) and in the discussion, "This conclusion was made based on our comprehensive analyses of Hp cytoarchitecture and gene expression patterns across the entire Hp in the COUP-TFI mutants. This whole-Hp analysis approach enabled us to capture phenotypes that might have been missed by examining marker gene expression on individual sections." (pg.13)

The upregulation of other dHP markers by qPCR from whole HP in the cKO is not very informative, since it may also reflect an overall abnormal differentiation and misregulation of these genes across the HP rather than the specific expansion of the dHP. Showing upregulation and expanded expression of multiple dHP markers in the tissue via spatial transcriptomics or in situ hybridization will better support this point.

OUR RESPONSE

We agree with the reviewer that changes in the expression of a few genes could potentially result from their misregulation. To address this concern, we have incorporated additional marker analyses using both qPCR and in situ hybridization (see response below) to better clarify the observed phenotypes. We now used the expression of Rbfox3 (NeuN) gene to assess neuronal differentiation and found no significant differences across genotypes (in new Figure 2H). Importantly, we found correlated changes in the expression levels of dorsal- and ventral-enriched genes in COUP-TFI mutant Hp (new Figure 2H and 3B), supporting a broader patterning effect rather than isolated gene misregulation.

Given the discrepancies in Wsf1 expression with the literature, the claim regarding the "expansion of dHP" needs to be further supported by showing expression in situ of additional markers that distinguish dHP and vHP. Perhaps using the same markers as in the previous studies will help to clarify the starkly different results across studies, in addition to the use of new markers which will add to the rigor and strength of the data.
Similarly, the support for the claim that the vHP is ventrally shifted concomitant with the expansion of the dHP is unclear. Their claim that Dcn (vHP marker) expression is shifted ventrally is unclear based on the images in Figure 2D, and not supported by Dcn quantification in Figure 2F, which does not show statistically significant differences in Dcn between the cKO and the control. The authors showed reduced expression of other vHP makers by qPCR from the whole HP, which as explained above, only indicates an overall reduction in the expression, but is not useful to support the claim that the vHP has shifted ventrally, has become smaller, or any positional effect.
OUR RESPONSE
As shown in the previous figure, we believe the discrepancy between our study and previous studies stems from differences in data interpretation rather than the choice of marker genes. In fact, we used the same dCA1 marker, Wfs1, to define dCA1. In this revision, we have included a new figure (new Figure S7) with in situ hybridization of additional vCA1 marker genes to demonstrate the vCA1 is shifted ventrally in the cKO. Additionally, for Figure 2F, we have increased the sample size and now showed a statistically significant difference in Dcn between cKO and control.

Overall, the authors are pushing the conclusion that the balance between the regions specified as dHP and vHP is shifted in the Couptf1 cKO favoring the expansion of dHP. Though the premise is interesting, the data presented are currently insufficient to support that conclusion. Stronger data (showing the expansion of expression in situ of several makers that unequivocally distinguish dHP and vHP) is needed to claim that the territory demarcated as dHP has acquired the correct molecular identity of dHP and that the region is indeed expanded.

OUR RESPONSE
Thanks for the reviewer's suggestion. We have added additional markers in Figure 2H and 3B S7.

In addition to the examination of molecular markers distinguishing dHP and vHP, the authors mentioned the distinct connectivity of dHP and vHP with other structures such as the cortex, amygdala, hypothalamus, etc. The authors could use retrograde tracing from different structures to identify the dHP and vHP regions, and determine their identity based on connectivity as an additional method.
OUR RESPONSE
Thanks the reviewer for this valuable suggestion. We did consider performing connectivity-based experiments to distinguish between dorsal and ventral Hp. However, we encounter a problem—the cortex itself is mispatterned in the COUP-TFI mutant mice (Armentano et al., 2007 and Feng et al., 2021). Thus, we feel it will be difficult to interpret the connectivity results and these experiments would not lead to insightful conclusions.

2.Location of CA2, and boundary between CA1, CA3 doesn't seem to change in Couptf1 cKO compared to control.
The authors show that the expression of Pcp4 (CA2 marker) is detected between CA1 and CA3,

similarly in cKO and controls (Figure 3A), and state "In the cKO, a Pcp4+ domain was detected between CA1 and CA3, similar to the control". If the relative position of CA2 is similar in Couptf1 cKO and controls, how do the authors reconcile these data with their conclusion regarding the dorsalization of CA1 and the expansion of the dCA1? Shouldn't CA2 be affected/shifted too?

OUR RESPONSE

Due to the lack of dorsal- or ventral- specific CA2 markers, we were unable to determine whether COUP-TFI is involved in the dorsal-ventral patterning of the CA2. We added this point to the discussion on Pg.15.

3. Couptf1 regulation of Wnt downstream genes. This is a potentially interesting mechanism revealed by the authors' ChIP-seq data. They suggest that Axin2 and Lef1 expression is regulated by Couptf1 in the neuroepithelium. In support of this claim, they show the expression of Axin2 and Lef1 by in situ hybridization. Images illustrating changes in Lef1 expression level between cKO, cTG, and controls are convincing, though it is unclear how the authors quantified protein expression level in Figure 4D, as claimed in the text. The images presented in Figure 4C illustrating Axin2 expression changes are less convincing and there is no quantification of Axin2 expression. Clarification on the quantification of expression, and perhaps additional data supporting these changes in expression would strengthen this interesting claim.

OUR RESPONSE

We thank the reviewer for the comment. We have selected more representative images for Axin2 for Figure 4B and performed quantification of both the Axin2 and Lef1 expression gradients. The results are now presented as a heat map in Figure 4D.

Minor comments:

1. Incomplete header: "COUP-TFI expression levels regulate the position of CA2 and the dorsal-ventral balance in CA3 and"

OUR RESPONSE

We thank the reviewer for catching this error. We have made changes to the header.

2. The statistical tests applied in each figure showing quantitative comparisons are not described in the figure legends.

OUR RESPONSE

We thank the reviewer for this comment. The statistical tests have now been described in the figure legends.

3. The method lacks detailed description and rigor. There is no methodological description for "protein quantification" for Couptf1 and Lef1 described in the text corresponding to experiments presented in Figure 4D. What kind of protein quantification method was performed?
The 'Stereological analysis, Image acquisition, and Quantification' methods section is poorly described. It seems from their description that the authors performed their volumetric analysis using planimetry, not stereology. If stereology was indeed used, they need to state the probe they used for area estimation and provide the error coefficient for their estimation. It is important to state at which magnification the images were acquired. This influences the accuracy of measurements and quantification.

OUR RESPONSE

We have now included the following description in the Methods section to clarify the quantification performed for Figure 4D: "To determine gene expression gradient (Fig 4D), HNE was divided into six equally spaced domains on five adjacent coronal sections. The average intensity of RNA in situ hybridization color reaction signals was measured within each domain. These intensity values were normalized to the background level of each section. Relative expression levels were then calculated by comparing the average intensity of each domain to the highest expression observed, which was in the most caudal ventral domain. The average intensity values of each domain was then visualized as a heatmap to illustrate the gradient." Additionally, we thank the reviewer's suggestion and have removed the term "stereological analyses" from the revised manuscript.
